# Physicians have feelings: illuminating the relationship between emotional valence, clinical reasoning and context specificity

**DOI:** 10.1080/10872981.2024.2404299

**Published:** 2024-09-23

**Authors:** Thomas Bertagnoli, Steven Durning, Michael Soh, Jerusalem Merkebu

**Affiliations:** a Department of Pediatrics, Uniformed Services University of the Health Sciences, Pediatrics, Bethesda, MD, USA; b Center of Health Professions Education, Uniformed Services University of the Health Sciences, Bethesda, MD, USA

**Keywords:** Medical education, clinical reasoning, situated cognition, Emotion, Valence

## Abstract

**Introduction:**

Research demonstrates that emotions play an important role in clinical reasoning (CR); however, the relationship between emotional valence, CR, and the context in which reasoning takes place, remains to be empirically explored. While situated cognition has been used to investigate CR and context specificity (e.g. the presence of contextual factors, things other than the information directly related to establishing a diagnosis), it has not explicitly examined the role of emotional valence during CR encounters. Our research question was how do emotional valence and arousal emerge in CR, particularly in the presence or absence of contextual factors?

**Methods:**

Physicians (*n* = 45) reviewed two video cases, one with contextual factors and one without. Immediately afterwards, participants completed a ‘think-aloud’ while reviewing cases. Thematic analysis was used to code transcribed think-alouds for CR activities, emotional valence (positive, neutral or negative) and arousal by three researchers. Frequencies and relationships between codes were compared, both in the presence or absence of contextual factors.

**Results:**

The majority of emotional valence codes were neutral (85.2%), with negative valence more frequent (11.2%) than positive valence (3.5%). Five CR themes were consistently demonstrated: knowledge organization (with two sub-themes of linking and differential diagnosis formation), proceeding with caution, curiosity, assumption, and reflection. In the presence of contextual factors, there was an increase in negative valence with a decrease in positive valence, as well as a shift in CR from knowledge organization to curiosity and proceeding with caution.

**Discussion:**

The complex interaction between clinical reasoning themes, emotional valence, and changes with contextual factors have important implications for clinical practice, education, and future research on CR.

## Background

Clinical reasoning (CR), which has been defined as the cognitive activities where a physician gathers and synthesizes information, generates hypotheses, and formulates a diagnosis, prognosis, and/or management plan [1], is a fundamental ability in the practice of medicine [2]. The literature has primarily focused on cognitive activities that lead to sound CR, which optimally should translate to safe and high-quality patient care. Recently, the importance of affective, or emotional, components of CR has garnered attention – namely that we are affective beings rather than unfeeling processing machines, and therefore should not conceptualize CR as a pure cognitive effort. This is congruent with the trend of understanding the impact of emotions in decision-making in the broader literature [3,4]. In a recent literature review, emotion has been shown at times to reduce cognitive fixation and enhance attention; conversely, it can introduce bias or impede cognitive processing, which can directly impact patient safety. Due to this variation in the literature, the review suggests that a clear relationship between emotion and CR has not yet been determined [5], making it difficult for medical educators to teach learners how to acknowledge their emotions and utilize them appropriately for effective CR. Developing this emotion-oriented approach to teaching CR is in stark contrast to previous reviews focused solely on how to teach the cognitive portions of CR [6]. If we desire to teach medical learners how to reason effectively (e.g., in the presence of inevitable environmental distractors or contextual factors), we must empirically explore the intricate interaction therein – utilizing both emotion and cognition to define this relationship with greater clarity.

Situated cognition is a theory that has helped us understand the complex nature of clinical reasoning which is dependent upon both content (e.g., case specificity) as well as context (e.g., situation to situation specificity). Contextual factors, whether they be physician, patient, or encounter/environmental, are things other than information that are directly tied to establishing a diagnosis (e.g., patient challenges the credentials of the physician or the electronic health record malfunctions during an appointment) and have been found to play a primary role in context specificity [7]. The effects of contextual factors on CR have been studied extensively, demonstrating how they can increase cognitive load, experts can mitigate the effects of contextual factors by awareness, and medical students are more susceptible to contextual factors [8–10]. Recently, studies employing this theory suggest that emotion could influence CR, but emotion has not been explicitly studied in the situated cognition approach to CR to date. For example, one study by McBee et al. [11,12] focused on the impact of contextual factors on CR of resident physicians, showing contextual factors increased diagnostic uncertainty and cost of care. This study did note contextual factors created emotional reactions in the residents but did not elucidate how this may have affected their CR. Another study utilizing military physicians showed cases with contextual factors brought forth more emotional language compared to those without [7]. In sum, these studies suggest that situated cognition may be an important lens that helps illuminate the relationship between CR and emotion through exploring specific contextual factors, but no studies have explicitly explored this yet.

One important theory for understanding emotion in health professions education is valence theory which posits emotion has two vectors – valence and arousal [13,14]. Contemporary valence theory refers to the spectrum from negative to positive feeling of that emotion – with a degree of neutrality between these two poles. Neutrality has addressed previous concerns about the oversimplicity of dichotomizing emotion to positive or negative aspects alone [15,16]. Arousal can be activating or deactivating; when applied to CR, emotion present either activates, or promotes, further contemplation and thinking through the problem at hand, or it deactivates and thus shuts someone down from continuing to think about and reason through a problem.

While the theory of situated cognition, in the presence of contextual factors, posits emotion’s potential role in CR, studies that help us understand the relationship between emotional valence and CR, which may be a leading mechanism resulting in context specificity, are lacking. To generate a better understanding of the relationship between emotional valence and CR, we qualitatively explored think-aloud transcripts with contextual factors to enhance our understanding of context specificity. Thus, our research investigated how do emotional valence and arousal emerge in CR, particularly in the presence or absence of contextual factors?

## Methods

We conducted an IRB approved study of residents and attending physicians in internal medicine. Participants viewed outpatient clinical video cases, one case with a contextual factor and the other without. After viewing the video, the participants reviewed the same videotape encounter using a standard think-aloud procedure facilitated by a research assistant [17]. We used videos as they represent an optimal way to conduct this investigation as videos (widely used training tools) ensure all participants receive an identical ‘stimulus’ to fully control both case content (identical content provided) and potentially relevant contextual factors (e.g., to empirically explore what may underpin context specificity). The transcripts we focused on were of an unstable angina case; the distracting contextual factors, if present, included the patient being concerned about a particular diagnosis that was not the actual diagnosis, as well as additional clinical information that distracted from the primary diagnosis. The videos lasted from just under 4 to 6.5 min and portrayed a clinical interview, a brief physical exam, and still screens of laboratory findings” [7]. In total, we had 45 participants’ think-aloud transcripts to analyze. Participants were a mix of PGY-1 interns, PGY-2 or PGY-3 internal medicine residents or attending physicians from Uniformed Services University and Walter Reed National Military Medical Center who were recruited by email invitation. Along with transcripts was demographic data of participants, to include gender, age, and level of training.

Initially, we performed an iterative inductive qualitative analysis of the think aloud data coding at the level of utterance, focusing on identifying clinical reasoning activities that were being performed. Subsequently, data was coded using standard content analysis techniques [18], creating a codebook where these CR activities were identified and then evaluated for emotional valence – either positive, neutral, or negative – and language was analyzed to determine if that activated further thinking on the case or if it led to deactivation to stop thinking about the case. In parallel, similar to Fereday and Muir-Cochrane [19] who demonstrate the rigor of hybrid techniques, the progression from individual codes to overarching themes was achieved through a combination of inductive and deductive approaches. The codebook was refined by iteratively establishing consensus on the analysis of shared transcripts and extracting exemplar quotes.

### Coding

Initially, five transcripts were reviewed and coded for possible clinical reasoning activities that were refined through regular meetings and iterative refining as we continued to analyze the transcripts. Through these meetings and discussions, we identified that the CR activity codes were actually themes, and linking and differential formation were sub-themes of knowledge organization. Consistent with the valence theory of emotion and similar to Merrill et al. [20] utterances were coded for emotional directionality to one of the three types of valence: positive, negative, or neutral and to one of the arousal categories: activating or deactivating categories [13,15]. To establish inter-coder reliability, the second and third researchers (MS, JM) each coded 10 (22%) of the think-alouds and then the entire study team reviewed the codes and utterances until consensus on CR activities, valence and arousal was achieved. The research team met to consistently analyze, interpret, and synthesize the results [21].

Additionally, to expand upon these findings, similar to La Pelle [22] we quantified the frequency of code instances to compare average code per transcript in the presence or absence of contextual factors (in combination) to inform themes that emerged inductively. We did not use inferential statistics given our sample size.

### Integration

In order to integrate the CR emergent themes with the valence theory, in the presence and absence of contextual factors, we employed Moran-Ellis et al. [24] following a thread. This analytic

integration approach consists of “identifying a ‘promising’ finding within a data which could be picked up as a thread to be followed through into the other codes [25]. When utilizing multiple theoretical frameworks, a promising emergent code may be identified by the relationship between the overarching research question and by the resonance of it with one or more of the other data [25]. In this respect, following the thread was particularly valuable as it enabled us to gain intensive insight into the interdependence of the clinical reasoning codes, emotional valence, arousal (activation/deactivation) situated across contexts.

### Reflexivity

Our research team recognizes that our training and experiences impacted our research choices and the conclusions we presented. Two of the team members are physicians (TB, SJD) who contributed to reflections around clinical reasoning (considering both the affective and cognitive dimensions) from their respective practices and the extant literature. TB is a general pediatrician who has served both in clinical and academic positions. JM is an educational psychologist in health professions education with expertise in metacognitive reflection, emotions, emotional regulation, and mixed-methods designs. Her background informed the theoretical framework, methodology, and interpretation of the data. MS is an educational researcher in health professions education who has studied CR and emotions and has expertise in both quantitative and qualitative methodologies. SJD is a senior researcher and an internal medicine physician; who has studied CR and context specificity for over a decade. Our diverse medical education research team, dedicated a tremendous amount of time to dissecting and debating the cognitive and affective boundaries of CR (grounded in the literature and drawing from experiential knowledge) to guide our interpretations and move the field forward.

## Results

Forty-five individuals participated in this study and the majority of emotional valence codes were neutral (85.2%), with negative valence present more often (11.2%) than positive valence (3.5%). The vast majority of utterances were activating (94%) with minimal deactivation (6%). Notably, in the presence of contextual factors, there was an increase in negative valence and deactivation, and a decrease in instances of positive valence. Five CR themes were identified: knowledge organization (with two sub-themes of linking and differential diagnosis formation), proceeding with caution, curiosity, assumption, and reflection. Below we present a detailed discussion of the CR themes, in relation to valence and arousal in the presence or absence of contextual factors. Summary of key frequencies of codes and demographic information for participants is found in Tables 1 and 2 (which displays how the average codes emerged per transcript and is discussed in greater detail below).

**Table 1.** Demographics.

Demographics	
Gender	N
Male	32 (71%)
Female	13 (29%)
Age	Avg 35 yrs
	Range 26–67 yrs
Level of Training	N
Intern (PGY-1)	12 (27%)
Resident (PGY-2/3)	12 (27%)
Attending	21 (46%)
Contextual Factors (CF)	N
Present	20 (44%)
Absent	25 (56%)

**Table 2.** General coding trends.

Characteristics	
Code Count	Avg per Transcript:
Total	20.1
With CF	23.7
Without CF	17.4
Valence	Avg per transcript
Neutral	17.2 (85%)
Negative	2.3 (11%)
Positive	0.7 (4%)
Activation	Avg per Transcript
Activating	18.9 (94%)
Deactivating	1.3 (6%)
Clinical Reasoning Theme	Avg per Transcript
Knowledge Organization	10 (50%)
Linking	5.9 (60%)
Differential Formation	4.1 (40%)
Caution	3.7 (18%)
Curiosity	3.2 (16%)
Assumption	1.6 (8%)
Reflection	1.6 (8%)

### Valence

The majority of the emotional valence codes were neutral (85.2%), with negative valence present more often (11.2%) than positive valence (3.5%). Negative valence tended to center around utterances evoking either worry for the patient or frustration at either the patient or the video physician: *“But when he puts his hand on his chest, that’s always distressing, um, just ‘cause it’s – it’s just a sign of cardiac issues”* (JPC027) *‘This is what we call a poor historian, where he’s just throwing out kind of extraneous information’*(JPC016). Conversely, positive valence clustered around utterances of relief when the participant interpreted information to suggest a less worrisome diagnoses or praise towards the video physician: *‘Taking some antacids and having the pain relieved is probably a pretty good sign that it’s reflux’* (JPC-I-004) *‘That’s good that he’s thinking about a PE, it’s always good to consider’* (JPC-I-002). Notably, while contextual factors did not affect neutral valence, an increase in negative valence (78.6%) and decrease in positive valence (38.6%) was observed in physician think-alouds (Table 3).

**Table 3.** Effects of contextual factors.

	w/o CF (codes/transcript)	w/CF (codes/transcript)	% Change (compared to overall change in total codes)
Total Codes	17.4	23.7	Baseline
Reflection	1.3	2.1	+10.7%
Caution	2.9	4.7	+15%
Curiosity	2	4.8	+76.4%
Assumption	1.4	1.9	−2.9%
Knowledge Organization	9.5	10.6	−20%
Linking	5.8	6	−25.7%
Differential Formation	3.7	4.6	−11.4%
Neutral	15	20	−4.3%
Positive	0.8	0.7	−38.6%
Negative	1.4	3.4	+78.6%
Activating	16.4	22	−4.3%
Deactivating	0.7	2.1	+103.6%

### Arousal

The vast majority of codes were activating (93.5%) as participants clinically reasoned. When deactivation occurred, it tended to either be present in utterances where it appeared the participant was giving up on figuring out what was going on with the patient or starting on a tangent thinking about other things than the case itself: *‘So, I don’t even know what differential to come up with. Now you’re dealing with multiple complaints. Multiple differentials for each one … it’s all becoming a big, fat blur’* (JPC016). In the presence of contextual factors, deactivation was notably increased (103.6%) while activation was not impacted (see Table 3).

### Knowledge organization

Knowledge organization was the dominant theme accounting for 50% of CR codes and was defined as the process where participants weave data points together with their fund of knowledge and prior experiences to build arguments for possible or definitive diagnoses. Knowledge organization was broken into two related sub-themes: linking and differential formation. Linking occurred more often than differential formation, comprising 60% of knowledge organization.

The goal of *linking* in CR was to take individual data points and begin adding them together to build an argument towards a conclusion. Participant JPC-I-004 states *‘… it was kind of a burning pain, woke him up this morning. Yeah. Taking some antacids and having the pain relieved is probably a pretty good sign that it’s reflux.’* The participant links the characteristic of pain with its timing and relief by antacids to arrive at a conclusion that the patient is suffering from reflux. Linking was obvious when the participant listed out individual data points explicitly, but sometimes participants implicitly refer to previous data from earlier in the transcript: *‘So he’s got an exertional component to it, which again, makes you think more cardiac, less likely pulmonary, although possible. And he’s a middle-aged guy, so that makes good sense for him’* (JPC025). This example also highlights how the conclusion of linking can be definitive while promoting open-mindedness to other possibilities.

Conversely, differential formation attempts to take available data and consider all the possibilities that could be: ‘*so you start thinking about things that could happen in the chest. Could be reflux; could be heart disease’* (JPC025). Here differential formation originated from a single data point: the location of the chief complaint. At other times, it came from more than one data point, where the participant used burning pain without distress to consider both heart attack and heartburn as dual possibilities: *‘It’s reassuring that it doesn’t look like he’s in distress and that he’s saying it is burning, although that doesn’t quite rule out a heart attack. So, it sounds like he’s describing heartburn now, which again is reassuring’* (JPC-I-002). Here, differential formation possibilities were explicitly noted, but at times a new possible diagnosis was listed for the first time. The end result of differential formation was to ensure *all* possibilities are considered and none are ruled out too soon.

When separating linking and differential formation as prominent subthemes, some unique features emerged. Linking had the second lowest proportion of deactivation – adding data towards a conclusion may sound like a process that excludes deactivation by definition, but linking could become deactivating when the participant began to think more about their thinking process rather than working through the case: *‘So I try to take that individual piece of information, and try to piece it together with other information they’ve given me so far’* (Table 4 h) or the conclusion they arrived to brought their reasoning to a quick stop: *‘So heart disease in 80s, not really that contributory. Family member with heartburn is very common’* (JPC-I-004). Differential formation had the lowest proportion of deactivation; the only time it emerged was in a rant where the participant takes the information presented so far and concludes there are so many possible answers that there is no way to determine what is going on and they gave up. *‘So, I don’t even know what differential to come up with. Now you’re dealing with multiple complaints. Multiple differentials for each one. Shitty history taking and it’s all becoming a big, fat blur.’* (JPC-I-004). Notably, differential formation also had the second highest proportion of positive valence, appearing when participants were relieved that the data pointed to a less concerning diagnosis.

In the cases with contextual factors, there were more utterances focused on clarifying the additional information provided while simultaneously trusting some of the presuppositions the patient presented about their own conclusions about their diagnosis. This resulted in decreased overall knowledge organization (−20%), the majority of this effect coming from a greater decrease in linking than differential formation (Table 3).

### Proceeding with caution

Proceeding with caution was the second most common theme, representing 18.2% of CR codes. Proceeding with caution was defined as a CR activity where participants verbalized not wanting to ascribe too much meaning to a data point, or they were uncertain about the relevance of a datapoint to their line of thought. For instance, JPC044 states *‘Looking at him he seems obese to me, but I don’t like to judge that based on … I want to see height and weight*.’ Even though their visual assessment of the patient makes the participant think the patient is obese, they remain cautious of that interpretation until definitive measurements are obtained. The degree of caution could vary from minor caution to explicit uncertainty: ‘*… it’s also important. I just don’t know what it is yet*.’ (JPC016).

Proceeding with caution had the second highest proportion of deactivation and notably, the lowest proportion of positive valence – occurring once. Caution appeared to promote cognitive paralysis and prevent participants from further exploring the case. For example, JPC016 was initially trying to determine if GERD compared to a cardiac cause, but as more and more information emerged that did not fit the original hypotheses, they become more and more cautious about interpreting facts until they gave up trying to solve the case: *‘It’s just like … what does all this mean?’*.

Approximately one-third of the time proceeding with caution appeared in tandem with another CR theme. For instance, proceeding with caution was frequently paired with curiosity, like in the obesity quote above where the participant is cautious about their visual assessment of BMI and pairs that with curiosity about height and weight to confirm their suspicions. It was paired with linking when trying to add pieces of the data together towards a conclusion, but understood it was a tentative rather than certain conclusion as evidenced here: ‘*… it was kind of a burning pain, woke him up this morning. Yeah. Taking some antacids and having the pain relieved is probably a pretty good sign that it’s reflux’* (JPC-I-004). Participants use language like ‘pretty good sign’, showing a relative degree of uncertainty in their conclusion. Proceeding with caution frequently paired with differential formation and also appeared in tandem with assumption as the participant understood those assumptions must be taken with a measure of uncertainty.

The presence of contextual factors was associated with a 15% increase in proceeding with caution, where additional information from the patient given alongside the patient’s presuppositions about their diagnosis caused the participants to slow down and question the relevance and relationship of the data to the case as a whole (Table 3).

### Curiosity

Curiosity was the next most common theme and was defined as the CR process where participants sought new or additional information they felt was missing, or requested clarification on information already presented because they felt there was more to be gained at that time. JPC055 demonstrates the desire to know new additional information in this quote: *‘So when he’s talking about his past medical history, I feel like, again, I just need more information about this type of stuff that, what does he take for his high blood pressure, how often does he take it, has he actually taken it today, what was the concern for the early onset diabetes, did they say that he had pre-diabetes, did they say that they thought he had, you know, needed to control his diet, what exactly happened in that situation?’*, while JPC029 demonstrates the clarification aspect by stating *‘So you know I think he told us again, a lot of good information but, I’d want to know more about like each specific piece of information at this point*.’ The first aspect of curiosity was generative – a sign or symptom started a thought process, but because the video physician did not continue in that line of thought, the participant hungered for more. The second was confirmatory – once the information pointed in a certain direction, the participant wanted just a little more to ensure they had come to the correct conclusion.

Curiosity’s valence pattern was notable for the second lowest proportion of positivity, instead remaining neutral or having negativity like in this quote: *‘So I’m a little bit frustrated with the examiner at this point because he doesn’t continue along that line of reasoning. He asks an open-ended question and when he doesn’t get the information he wants, he just moves on to ask about when it started, whereas I have about 75 more questions I want to ask; slight exaggeration, probably 5 or 6’* (JPC055). Curiosity also had the second lowest proportion of deactivation. Wanting to know more information about the case was by definition activating; therefore, curiosity could only be deactivating when the participant wanted to know more information about something not directly related to the case as when JPC055 when on a tangent about wanting to know more about the units of measurement for calcium levels and lab normals.

Curiosity did not occur in tandem with other themes as often as others; when it paired, it frequently occurred with proceeding with caution. In some ways, curiosity and caution may seem like opposing themes: proceeding with caution slows cognitive momentum to ensure not misinterpreting information, while curiosity stokes cognitive momentum in wanting to know more. However, the first quote we viewed demonstrated how they work together: the participant had caution in interpreting the limited and biased history the patient presented, and that caution triggered curiosity to know more and clarify the information presented.

Contextual factors appeared to impact instances of curiosity (+76.4%) (Table 3).

### Reflection

Reflection was the second least common theme, defined as the CR cognitive activity where a participant contemplated previous experiences and/or medical knowledge with the purpose of processing and interpreting the data currently being presented to them. Sometimes reflection could be on immediate experiences – the last patient seen or an event earlier in the case. Others could be based on more distant experiences – previous tests or simulations or other similar cases. Some were not as direct, reflecting on how different approaches affected obtaining and processing information or how one might do better if they had to do it again. For example, JPC041 demonstrated several types of reflection. First, the participant reflected on how the physician’s communication style led to insufficient information and a poor therapeutic relationship when they said ‘*So, I don’t like how the doctor kind of cuts him off there and doesn’t address that concern. If nothing else, just like acknowledge it and kind of like yeah think about it or just like make him feel heard because he just kind of gets cut off there’*. Later they reflected on how the exposure history does not match a predefined illness script when they said ‘*So, yeah the cough being persistent since the coal mine, it’s just weird. Like typically if you have like exposures or something, you know, some sort of like black lung disease or something like that, you would expect that to kind of develop over time. Of course, we don’t know how long he worked in the coal mines’*. Finally, later on in the case they reflected on how lab and imaging results are different from what was initially anticipated ‘*I shouldn’t have put CHF because really you, yeah, with a clear chest x-ray like that, it’s not CHF. So, yeah, that was dumb.’*

Reflection was typically activating, as long as it remained focused on the case; however, it had the highest proportion of deactivation of all the CR themes. JPC041 also demonstrated this well, where the reflection started to pull them down a rabbit trail to focus on something besides the case at hand: ‘*I was just kind of laughing because he is a – it shouldn’t be funny I guess, but you often get patients that are like, you know, do you smoke and they’re like, “No,” and then when did you quit? Yeah, a week ago or today or yesterday and it’s like oh, okay, you’re smoking. So, yeah, I would consider this guy an active smoker, but it’s funny how he is kind of in denial about things like oh, yeah, I quit and has this great story to tell.’*

Reflection also stood out by having the highest proportion of positive valence. Positive valence developed because something about the patient or case reminded the participant of a fond or amusing clinical situation, like in the quote above, or because the data presented was reassuring considering the participant’s previous experiences.

The presence of contextual factors was associated with more instances of reflection (10.7%) (Table 3). For example, in the absence of contextual factors JPC032 only had reflection about what labs they missed the first time viewing the video, whereas JPC041 reflected on multiple different things in their case with contextual factors.

### Assumption

Assumption was the least common theme, defined as the process where participants fill in information they view as vague or nonspecific, not necessarily seeking to clarify if the data they filled in is accurate or not. JPC-003 provided an exemplar quote of assumption by stating *‘He was on a cruise. He ate – I was like, all right. So the food – usually you’re not having – you’re having a lot of oily, more spicy foods on a cruise, so type of food is different. So I was like, okay, GERD’* in their transcript. They took the datapoint of ‘recently on a cruise’ and assumed the patient would eat more spicy and oily foods but did not verbalize wanting to confirm that fact with the patient. Furthermore, these participants demonstrate that CR assumption does not need to carry a negative connotation. They employ illness scripts to describe common disease patterns, and as clinicians ‘fill in the gaps’ of information not directly obtained using illness scripts plus cues from the patient and the available information at hand. JPC025 did just that when they noted *‘…so prior smoker; so risk factor for both, uh, pulmonary disease and for cardiovascular disease. Okay, so it doesn’t say how old he is, but that’s probably a solid 30 years maybe, of use. That’s plenty to give him either one of those things. Okay, so older, not really early onset kind of history*’. Conversely, assumptions, if used inappropriately, carry the risk of inserting errant information, leading the clinician down the wrong path and ultimately leading to risk and medical error. This happened to JPC-I-003, who made multiple assumptions about the patient’s level of anxiety and smoking history, ultimately concluding the patient had GERD and/or COPD and missed the diagnosis of angina.

Assumption notably carried the second highest proportion of negative valence, as the most common assumption was about the pack-year smoking history with either explicit or implicit judgment against the patient for smoking so long.

Assumption had a unique relationship with curiosity; Participant JPC-I-003 initially demonstrated this when they assumed information about access to care and treatments previously tried but still wanted to know more about why the patient was nervous about the response to medication. They understood something was still missing despite the gaps they filled and wanted to know more to accurately understand the situation. Contextual factors did not seem to impact rates of assumption; in both cases, pack-year of smoking was not explicitly defined so the participants assumed a duration in both (Table 3).

**Figure 1.**
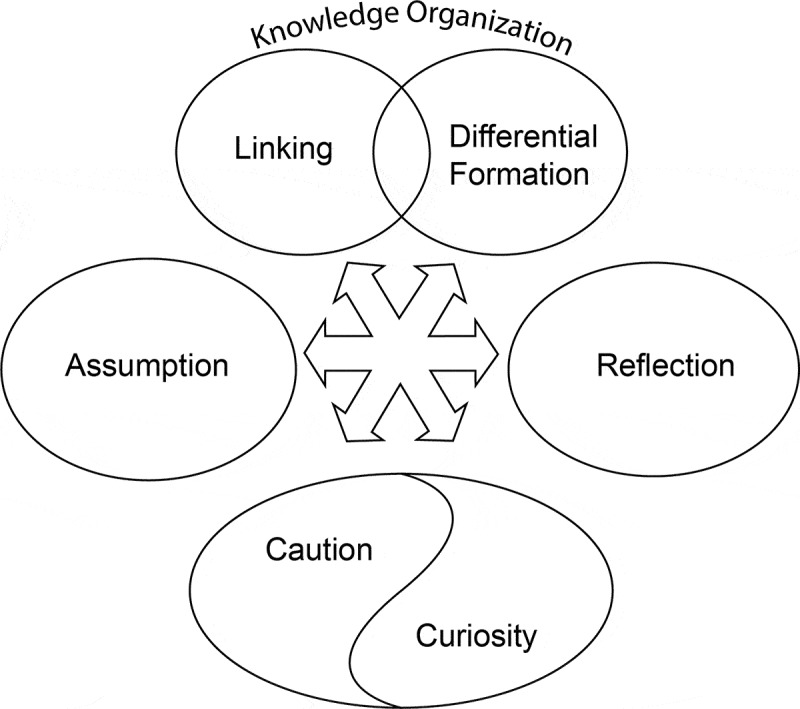
Concept map of relationship between clinical reasoning themes (figure created in adobe Illustrator).

**Figure 2.**
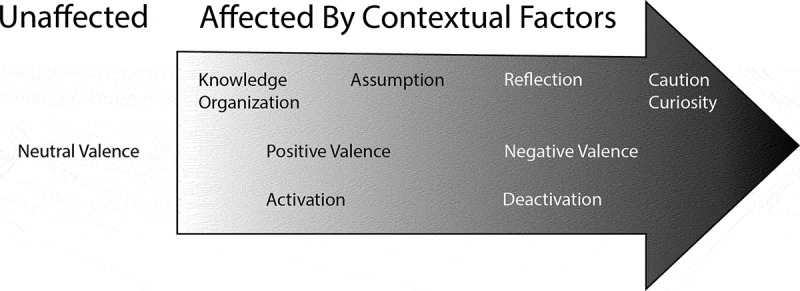
Effects of contextual factors on CR Theme, emotional valence and arousal (figure created in adobe Illustrator).

The concept maps (Figures 1 & 2) summarize the perceived interdependence of the aforementioned themes and the effect of contextual factors on CR themes, valence, and arousal, with a detailed discussion to follow.

## Discussion

The purpose of this study was to explore how valence and arousal emerge in the clinical reasoning process both in the presence and absence of contextual factors. Five interdependent CR themes emerged highlighting five distinct CR activities: knowledge organization, proceeding with caution, curiosity, reflection, and assumption; contextual factors shifted reasoning with a decrease in knowledge organization and an increase in curiosity and proceeding with caution. Although independent lines of research have discussed the impact of these constructs [2,26–28] to our knowledge, this is the first study to highlight and describe their interdependence in relation to CR and in the presence of contextual factors. Further, we discovered the majority of emotional valence was neutral; and negative valence was more frequently present than positive valence. Overall, contextual factors promoted increased negative valence and deactivation, while decreasing positive valence while leaving neutral valence unaffected. These results have important implications on clinical practice, education, and future research.

One might argue that the majority of emotional valence being neutral is equivalent to a lack of emotion, and therefore evidence that CR is a primarily a cognitive process; the fact that negative and positive valence are present proves that we cannot fully separate CR and emotion. We also hypothesize that a mixture of prior learning CR as a solely cognitive skill predisposed participants to discuss their reasoning in neutral valenced language, as well as the minimalistic think-aloud prompt ‘tell me what you were thinking’ predisposes neutral cognitive process language more than ‘tell me what you were thinking and feeling’ would. Similarly, when we break down the specific CR themes we see variations on proportions of negative vs positive valence, suggesting subtle differences in the emotional valence of those CR activities, even if most of the time they are described in neutral valence due to the two factors above. However, it is contextual factors that make notable changes to the emotional valence present overall, suggesting that these extenuating circumstances disrupt the tendency for neutrality, bringing to the surface the emotion even more of the emotion that was already present.

The complex interdependent relationships of the CR themes was an interesting finding that emerged as we followed the thread across the data. Their interdependence is consistent with previous research showing these diverse sets of relationships activate diagnosis, while eliminating incompatible options [27]. Organization of knowledge was a prominent component of CR but was not sufficient without support from the other reasoning themes. Early on in our coding and analysis, it appeared that linking would be the opposite of differential formation with linking solely narrowing possibilities, and conversely differential formation broadening them. However, as we reflectively analyzed the core content and meaning of the transcripts [29], we discovered that between a third to half of the time – linking and differential formation were occurring simultaneously, although possessing distinct goals. The result of linking in CR was to come to an answer of what they think is going on with the patient to determine the next steps. Conversely, the goal of differential formation was to think more broadly and ensure no possible causes have been missed. The overlap emerged when linking specific data points came to a new conclusion that had not been offered up as a possibility before, thus expanding the overall possibilities of what the clinical picture could be. This movement of generating a broad range of initial differential diagnoses and narrowing down as evidence emerges is consistent with previous CR research [2,30]. Linking and differential formation could be done in isolation, sequentially, or simultaneously; simultaneous pairings increased the efficiency of arriving at a diagnostic decision to take next steps in the patient’s care. When teaching medical learners CR, we may initially need to break down the steps of knowledge organization, but as learners progress, they must be taught the importance of combining these processes together; moving from scripted schemas to adaptively reframed metacognitions [31,32].

Every theme paired with a second theme at least once, and many utterances were coded with more than one CR theme, demonstrating that none of the themes exists in isolation and both clinical reasoning and emotion are incredibly complex, overall highlighting the need to account for these complex internal/external dynamics [30]. Proceeding with caution frequently occurred with knowledge organization as participants were reaching conclusions; they either had hesitance about their conclusions or kept minds open to further possibilities to avoid premature closure. Proceeding with caution and curiosity were also closely linked themes. The desire for accuracy and hesitancy to make mistakes, knowing they can lead to patient harm and bad outcomes, is a reasonable driver for caution occurring more frequently than curiosity. Often the hesitance the participant felt about the accuracy or usefulness of a piece of data drove them to want to clarify or know more to mitigate their hesitancy. Teaching this balance is important to avoid decision paralysis or committing to a conclusion without appropriate evidence. This is in line with CR research supporting the need to trust feelings but avoid jumping the gun incorporating both analytical and non-analytical reasoning [33]. Many times, we associate a negative connotation to assumption. However, in CR assumption does not need to carry a negative connotation or ultimately lead to error. We use illness scripts to describe common disease patterns, and a skilled clinician can therefore ‘fill in the gaps’ of information not directed obtained using illness scripts plus cues from the patient and the rest of information that is at hand [32]. Therefore, assumption interacted with proceeding with caution when a participant filled in some information, they also acknowledged their assumptions and therefore were cautious about assigning too much weight to their assumption. During our initial coding process, we posited that assumption may be the opposite of curiosity; rather than seeking to know more and clarify missing information, participants could fill in details and move on without seeking to ensure if their assumptions were correct. During our integration process, pairs of assumption plus curiosity occurred and proved they were not direct opposites. Mennin [34], highlights that “When learners are curious, they examine and question the assumptions about what they see and do at the edge of certainty and uncertainty, at the intersection of the familiar and novel, rich and generative learning emerges in medical education.

Contextual factors impacted CR themes as well as valence and arousal. Overall, there were more codes from transcripts with contextual factors, a trend that makes sense given the additional clinical information that included distractors and patient diagnostic bias. This is in accordance with prior work demonstrating context specificity, where the complex interplay of physician, patient, and environmental factors drive clinical reasoning and diagnostic error [35]. An increase in negative valence was not surprising as both the patient’s bias and additional data brought into consideration worrisome diagnoses like occupational exposure, cancer and life-threatening illnesses that resulted in concern about the patient’s diagnosis; also, the lack of organization and clarification on certain datapoints also lead to frustration in many of the participants. An increase in deactivation was also not surprising; it is hard not to get distracted and go off on a clinical rabbit trail when so much information is coming at you or to shut down when there is so much information without a clear picture of what could be going on. This is consistent with prior CR research demonstrating the negative impact of distracting contextual factors [33]. Consequently, when teaching CR, it is important for educators to nurture the development of metacognitive reflection via deliberate instruction [36] and practice that includes 1) metacognitive awareness of contextual factors in order that learners prevent deactivation and 2) emotional regulation of negative valence emotions, deactivation, extreme empathy, or frustration at the situation to clarify information and work towards a solution for their patients.

Shifting away from knowledge organization towards curiosity and proceeding with caution in light of the contextual factors present was an insightful finding. On one hand, one might think with more datapoints presented it would be easier to arrive at a conclusion or consider new possibilities; however, because many of the datapoints were added to the case without context or clarifying details, it’s plausible that participants had a hard time organizing and processing the additional information. As a result, participants proceeded with more caution about their conclusions and had additional questions they wanted answered to better understand what was going on with the patient. Richards et al. [37] also support that the ability to engage in effective clinical reasoning is influenced by curiosity. Additionally, contextual factors also increased reflection as participants thought back on past clinical experiences or their fund of knowledge to understand the patient’s situation better [23]. Clinical educators need to help teach medical learners how to balance the need to clarify and know more about organizing additional information towards actionable conclusions. Mennin [34], states ‘Promoting more effective learning requires attention to perceiving, understanding, and influencing how we set the conditions for learning’. As our findings indicate, understanding the complex interrelationships among valence/arousal, curiosity, knowledge organization, reflection, assumption, and contextual conditions are essential for learning/teaching effective CR.

### Limitations

Our study is not without limitations. While the think-aloud protocol allows for unprompted verbalizations from participants, the focus on asking about what they were thinking while viewing the case may inherently bias verbalization of cognitive processes rather than an in-depth interview that asks participants about their emotional experiences to what was occurring. Expanding the interview in this manner may introduce reference bias though, causing physicians to feel like they need to express emotion they did not feel at the time. Another limitation comes from utilizing only a single case and contextual factor. While we had enough qualitative data to reach thematic saturation, we cannot tell if our findings would be transferable to different case content or contextual factors: for instance, do surgical specialists have a similar relationship between emotion and their clinical reasoning? Do the contextual factors of diagnostic suggestion and large amounts of unrelated clinical information shift their reasoning and emotion the same ways? Further, due to our small sample size, we were unable to perform inferential statistics to assist in identifying statistically significant differences between code groups that may have elucidated subtle trends we did not discover during our analysis. Our population includes physicians from multiple levels, from beginning internal medicine residents to expert physicians, but we did not have enough in each group to separate out and see if different levels of experience experienced a different relationship between their clinical reasoning and emotion. Finally, our study was not able to delve into accuracy – do certain emotional valence or reasoning themes impact the accuracy of reasoning?

These limitations prompt a need for further study. One direction of study would be to use a similar study design on physicians from different specialties, such as general surgery, surgical specialties, emergency medicine, or pediatrics, to see if the relationship between clinical reasoning and emotional valence is similar or different. It is also important to evaluate if the relationship between CR and emotional valence varies between other groups – do different genders experience the same relationship? Do providers of different ethnicities reason with emotion the same way? Do military providers in a resource limited deployed environment reason with emotion the same way a civilian physician in a resource limited rural community health system would? Another direction to take would be to use the same analysis framework to other case think-alouds done in this program of research to see if different case specificity and context specificity change the relationship between CR and emotional valence. Our current findings already have an implication for health professions education: it is clear emotion is present and affects reasoning so we cannot teach CR as a solely cognitive process. Comparing the subtle changes in the relationship between CR and emotional valence at different levels of training may reveal areas of foci for educators to teach junior learners how to regulate and effectively manage their emotions. For instance, taking into consideration specialties that are often highly charged with emotion and examining how physicians psychologically construct or categorize CR-related emotional experiences [38]. Likewise, what emotions and emotional regulation strategies (proactive or suppressive) are likely to emerge among ER docs who are making clinical decisions about sickle cell patients or pediatricians counseling vaccine-hesitant parents on the risks of refusing routine immunizations? This interaction will allow us to gain a deeper understanding of the lived experiences of physicians, employing an interpretivist epistemological approach, to explore the phenomenon of CR within emotionally charged contexts. It would be a fruitful endeavor to simultaneously investigate if the physician’s capacity to effectively regulate their emotions has *any* (e.g., favorable) impact on the patient?

Employing methodological flexibility, the other avenue of research to pursue is tying in accuracy to this relationship – does the shift from knowledge organization to curiosity and proceeding with caution and increased negative valence ultimately detract from a clinician’s accuracy? Searching for and exploring patterns of CR, emotional valence, or arousal that might help predict diagnostic accuracy may inform and identify heuristics and other strategies that medical educators can use when teaching CR. By utilizing tools with validity evidence, like post-encounter forms (PEF) scores, in tandem with think-aloud data, we can better understand the negative and positive impacts of this relationship on accuracy. A research endeavor such as this could help educators develop interventions/strategies that help physicians optimally manage the negative impact of contextual factors, negative valence, and deactivation to promote diagnostic accuracy.

## Conclusion

Advancing research within this domain holds the potential to equip medical educators to teach learners how to harness emotion effectively – to deliver high-quality patient care. The current study discovered the majority of emotional valence accompanying CR activities was neutral; and negative valence was more prevalent than positive valence. Moreover, contextual factors promoted increased negative valence and deactivation (while decreasing positive valence). We encourage medical education researchers and practitioners 1) to develop metacognitive awareness of the valence accompanying their CR activities and 2) reflectively consider (proactive) emotional regulation strategies to manage negative emotions or up-regulate positive emotions [39]– in the presence of distracting contextual factors. We hope the findings of this study offer researchers a foundation to further clarify the relationship between CR and emotion and helps clinicians navigate the emotional landscape of clinical reasoning.
